# CHARACTERISTICS OF CHINESE ADULTS ON CANCER SCREENING COMMUNICATIONS WITH PHYSICIANS

**DOI:** 10.1093/geroni/igz038.680

**Published:** 2019-11-08

**Authors:** Su-I Hou

**Affiliations:** The University of Central Florida, Orlando, Florida, United States

## Abstract

This study examined characteristics of Chinese adults on cancer screening communication with physicians. Whether doctor recommended screenings and whether communicated screenings with doctor were used to assess cancer communication. Participants were recruited from 9 Chinese churches (5 in U.S. and 4 in Taiwan; N=372). Mean age was 44.31, 60% males, 72% married, 85% college education, and 54% had family history. Overall 35.2% reported doctor recommended screenings and 27.7% talked with doctors about screenings (27.7%). Regressions showed Chinese 40+ years (OR=2.66 & 2.49), had annual health exam (OR=3.43 & 4.41), and been a primary cancer caregiver (OR=2.12 & 2.29) were more likely to report doctor recommended screenings (p<.001; 69% correct classification) and communicated with doctors about screenings (p<.001; 76% correct classification). There were no significant relationships between family history, gender, perceived cancer risk or health, and screening communications. Findings have implication on designing effective doctor-patient cancer communication programs among Chinese adults.

